# “*Death Is a Possibility for Those without Shelter*”: A Thematic Analysis of News Coverage on Homelessness and the 2021 Heat Dome in Canada

**DOI:** 10.3390/ijerph21040405

**Published:** 2024-03-27

**Authors:** Emily J. Tetzlaff, Farah Mourad, Nicholas Goulet, Melissa Gorman, Rachel Siblock, Sean A. Kidd, Mariya Bezgrebelna, Glen P. Kenny

**Affiliations:** 1 Human and Environmental Physiology Research Unit, School of Human Kinetics, University of Ottawa, 125 University Private, Ottawa, ON K1N 6N5, Canada; etetz085@uottawa.ca (E.J.T.); fmour023@uottawa.ca (F.M.);; 2 Climate Change and Innovation Bureau, Healthy Environments and Consumer Safety Branch, Safe Environments Directorate, Health Canada, 269 Laurier Avenue W., Ottawa, ON K1A 0P8, Canada; melissa.gorman@hc-sc.gc.ca (M.G.); rachel.siblock@hc-sc.gc.ca (R.S.); 3 Slaight Family Centre for Youth in Transition, Centre for Addiction and Mental Health, 1001 Queen Street W., Toronto, ON M6J 1H4, Canada; sean.kidd@camh.ca (S.A.K.); mariya.bezgrebelna@camh.ca (M.B.); 4 Department of Psychiatry, University of Toronto, 250 College Street, 8th Floor, Toronto, ON M5T 1R8, Canada; 5 Clinical Epidemiology Program, Ottawa Hospital Research Institute, Ottawa, ON K1Y 4E9, Canada

**Keywords:** housing insecure, unhoused, unsheltered, public health, extreme heat, heat wave, heat alert and response systems, 2021 Heat Dome, Canada

## Abstract

Among the most vulnerable to the health-harming effects of heat are people experiencing homelessness. However, during the 2021 Heat Dome, the deadliest extreme heat event (EHE) recorded in Canada to date, people experiencing homelessness represented the smallest proportion of decedents (n = 3, 0.5%)—despite the impacted region (British Columbia) having some of the highest rates of homelessness in the country. Thus, we sought to explore the 2021 Heat Dome as a media-based case study to identify potential actions or targeted strategies that were initiated by community support agencies, individuals and groups, and communicated in the news during this EHE that may have aided in the protection of this group or helped minimize the mortality impacts. Using media articles collated for a more extensive investigation into the effects of the 2021 Heat Dome (n = 2909), we identified a subset which included content on people experiencing homelessness in Canada (n = 274, 9%). These articles were thematically analysed using NVivo. Three main themes were identified: (i) public warnings issued during the 2021 Heat Dome directly addressed people experiencing homelessness, (ii) community support services explicitly targeting this population were activated during the heat event, and (iii) challenges and barriers faced by people experiencing homelessness during extreme heat were communicated. These findings suggest that mass-media messaging and dedicated on-the-ground initiatives led by various organizations explicitly initiated to support individuals experiencing homelessness during the 2021 Heat Dome may have assisted in limiting the harmful impacts of the heat on this community.

## 1. Introduction

As the frequency, intensity and duration of extreme heat events (EHEs) have been increasing and are projected to continue to rise due to climate change [1], the impact of these events on individual and public health is a growing priority globally. In particular, it is well-established that EHEs disproportionality impact specific members of communities based on physiological factors, such as age [2] and chronic disease [3], as well as factors related to social and material marginalization (e.g., low income, people living alone, people living with insecure housing [4,5]). As such, there is a critical need for targeted strategies, policies, and public health interventions specifically designed to help address the unique challenges faced by those most heat-vulnerable to reduce adverse morbidity and mortality outcomes [6,7]. Among those most vulnerable to the health-harming effects of heat are people experiencing homelessness, including the unhoused and precariously housed [5,8,9,10]. However, to date, despite the growing attention to inequities in heat risk specifically, or extreme weather generally (e.g., cold, flooding), there has been limited academic and policy attention focused on the experiences, adaptations, and outcomes related to people who lack adequate shelter [11,12,13,14,15].

Homelessness can present in various ways and can reflect a range of housing situations, such as unsheltered/rooflessness (e.g., living on the street, emergency shelters), houselessness (e.g., living in various types of shelters or institutions), insecure housing (e.g., living under threat of eviction), or inadequate housing (e.g., residing in unfit or overcrowded conditions) [16]. During EHEs, those in unsheltered living situations are particularly at risk as they may be residing in public (e.g., sidewalks, parks) or private spaces (e.g., vacant buildings), or places not intended for permanent human habitation (e.g., cars, garages, attics, makeshift shelters, tents) [17], or other accessible non-airconditioned environments [11,18]. Further, based on the proximity to centralized supportive services (e.g., homeless shelters, food banks), people experiencing homelessness are more likely to congregate in areas of cities where temperatures are the highest due to the built environment (i.e., urban heat island effect) [10,19,20]. Compounding the vulnerability created by the lack of physical shelter from extreme weather impacts and exposure to the elements [5], this community also commonly experiences higher rates of pre-existing health conditions such as mental illness, substance use, and comorbid conditions (e.g., cancer, cerebrovascular, infectious diseases), as well as social isolation which can further exacerbate their heat susceptibility [9,10,14,21,22,23,24]. 

As a result of the complex challenges experienced by this population, employing optimal heat-mitigating strategies, such as operating air conditioning—the single most influential option for heat mitigation [25,26]—may not be feasible. Therefore, this heat-vulnerable group must often access community-based cool spaces (e.g., malls, libraries), formally established cooling centres, or rely on other publicly available resources, such as public water fountains, misting stations or splash pads [11,18]. Despite the efforts made in various regions to mitigate the health burden experienced by this population during EHEs, they have continued to be identified as a disproportionately impacted group based on increases in emergency department visits, hospitalizations, and heat-related deaths [5,13,27]. For example, Schwarz et al. [5] recently found that 10% of hospitalizations during heat waves in San Diego, California (USA), over seven years were people experiencing homelessness. Similarly, in 2020, 2.3% of the known homeless population of Maricopa County, Arizona (USA), died due to heat-related causes [13], and data from the United Kingdom has also demonstrated that unhoused populations are more likely to be hospitalized as temperatures rise than housed populations [27]. Therefore, as both extreme heat and homelessness continue to pose a public health challenge globally [7] and Canada has committed to addressing existing inequities and the climate’s disproportionate impact on marginalized and underserved populations [28], it is crucial to continue to investigate how different public health interventions address the specific needs of this heat-vulnerable group to help strengthen future extreme heat response initiatives. 

During the 2021 Heat Dome, the deadliest EHE recorded in Canada to date, 619 heat-related deaths were reported in British Columbia alone [29]. Of the decedents, people experiencing homelessness represented the smallest proportion (n = 3, 0.5%) [29]—despite this region having some of the highest rates of homelessness in the country [30] and outdoor temperatures exceeding 40 °C throughout the region during the heat event [31]. This stark difference in mortality compared to previous heat events globally [5,13,27] may indicate that some protection was present during the 2021 Heat Dome that might not be currently employed in other geographic areas experiencing heat-related deaths among this population. This finding, therefore, lends to inquiring about what actions or targeted strategies were initiated or communicated during the 2021 Heat Dome in Canada that perhaps aided in the protection of this group or helped minimize the overall mortality impacts. However, mortality data alone does not reflect the multiplicity of outcomes that can occur as a result of extreme heat (e.g., health deterioration, decreased physical and mental wellness, increased financial challenges, mobility impairment, impediments to social services [32,33]), especially in light of potential issues with detection and reporting accuracy among this population. Therefore, further investigation into the less understood implications of the 2021 Heat Dome on people experiencing homelessness is also needed [8,34,35,36].

Unfortunately, limited data sources are available on this population as the legal and social invisibility associated with homelessness often excludes them from conventional databases [37,38]. Further, no data sources currently collate the scope of services activated to serve this population during EHEs, except for media coverage. As with other extreme weather events, the 2021 Heat Dome drew significant news coverage from the first release of the heat alert by Environment and Climate Change Canada on 24 June 2021, and for several months post-event. Thus, we sought to explore this historic event as a media-based case study to identify potential actions or initiatives that may have supported people experiencing any form of homelessness during the 2021 Heat Dome, as well as explore the less understood implications of the heat event on this population. With more than 25,000 people experiencing homelessness on any given night in Canada [30], and increasing rates of homelessness being reported globally [39,40], the results of this study, considered in the context of current research on the subject, may help provide critical information on the heat–hazard context facing people experiencing homelessness [41,42], while giving evidence of the benefit of prioritizing additional institutional and individual response strategies within heat alert and response systems [43]. 

## 2. Materials and Methods

### 2.1. Search Strategy and Article Selection

This study is part of a larger research project that examines the portrayal and communication of health risks and impacts associated with the 2021 Heat Dome in Canada within online news articles [44]. In the context of this investigation, online news refers to any form of web-based media that delivered text-based content to the public digitally (except for social media), such as newspaper articles, radio broadcasts, and television transcripts produced by news agencies, newspapers, news magazines, and news channels. Therefore, all print media, content without verbatim transcription (e.g., audio and video-only content), and social media posts (e.g., Facebook, X, Instagram) were excluded.

The search strategy included a review of online media content from five subscription news databases (ProQuest Canadian Major Dailies, Business Source Elite, NewsDesk, Factiva, and Eureka). The search strategy was developed in consultation with a Research Librarian, and the final search underwent PRESS review before database translation. For all searches, the content was limited to English and French articles published within Canada between 1 June 2021 and 26 February 2022. A list of targeted public and non-profit organization websites (n = 997) was created for each province and territory in Canada, along with pan-Canadian sites and an Advanced Google search for key terms (e.g., “heat” and “2021”) to supplement the database searches.

The articles were uploaded to Zotero (Release 6.0, Corporation of Digital Scholarship), a digital citation tool. As some articles were released to multiple news sources (as media conglomerations share and modify content), similar versions of articles were identified, and deduplication was completed. French articles were identified and sent for professional translation. The translated documents were then checked for accuracy by a bilingual research team member (N.G.). The articles were then reviewed to ensure that the data captured included only Canadian-produced news articles and that the text primarily focused on the Canadian context. The resulting dataset for the primary analysis included 2909 articles.

**Figure 1:**
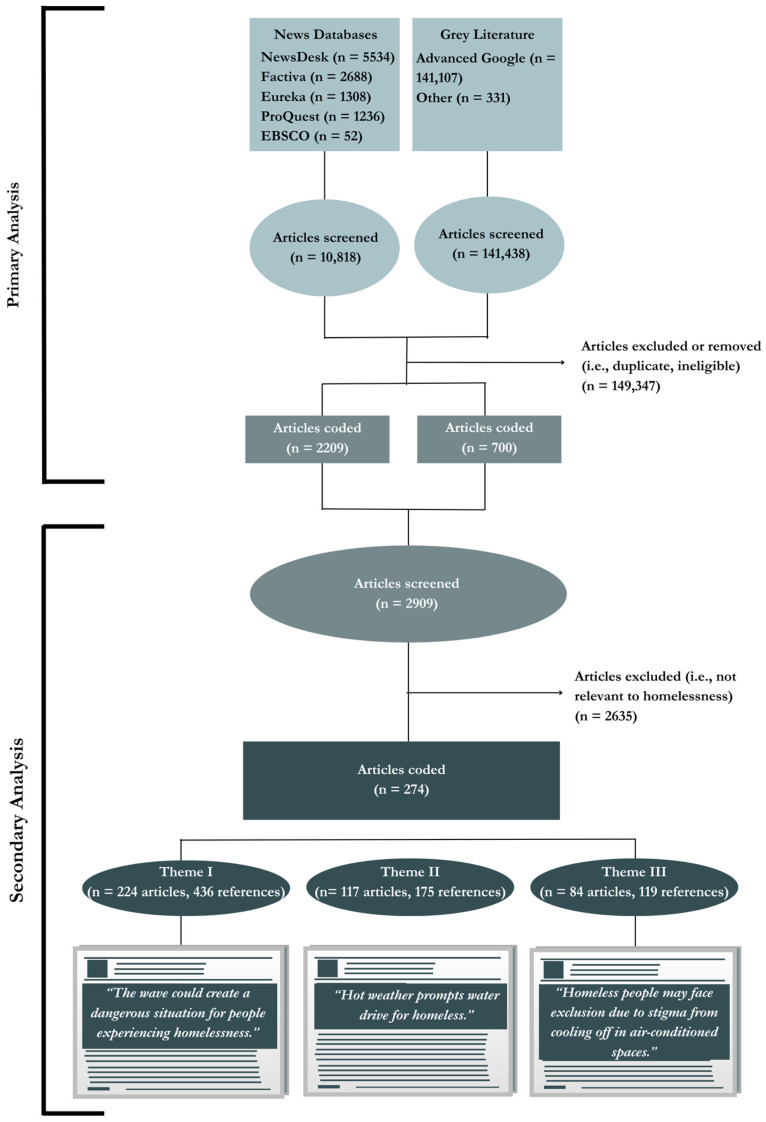
Flow diagram for the larger research project [44] and this secondary analysis, which focuses on people experiencing homelessness during the 2021 Heat Dome in Canada. Note: ‘n’ denotes the number of articles, and ‘references’ is the number of excerpts from the articles related to the theme.

For this secondary analysis, we then developed a list of key terms that would serve as positive indicators of content relevant to extreme heat and people experiencing homelessness. Terms indicative of homelessness were identified using the Medical Subject Headings (MeSH) index list, a controlled vocabulary thesaurus published by the National Library of Medicine. Terms included homeless(ness), houseless(ness), unhoused, unsheltered, and housing insecure, among others. The resulting dataset included 274 articles (9% of the larger dataset) (Figure 1).

### 2.2. Thematic Analysis

The data was then analysed using thematic analysis, which is a method that offers a way of identifying repeated patterns of meaning across a dataset [45]. Our analysis was theoretically underpinned by constructivism, with an inductive orientation allowing for the themes identified to be strongly linked to the data. We also took a semantic approach, which means that the themes we identified are based on the explicit or surface meanings of the data [46]. This approach aligns with our media-based dataset as it emphasizes the lens of the media makers themselves (e.g., journalists) in the initial interpretation of what is reported to the public [47]. We used a descriptive thematic analysis that prioritized how the media presented meanings and experiences in developing our themes [48]. This method is beneficial when investigating an under-researched area [48]. 

To conduct the thematic analysis, we followed the six steps outlined by Braun and Clarke [45] using NVivo—a qualitative content analysis software (Release 1.6.2, QSR International, Burlington, MA, USA). The two primary coders (E.J.T. and F.M.) initially familiarized themselves with the data during the coding of the larger project [44]; however, they then re-immersed via repeated active reading (i.e., reading while searching for meanings and patterns) of the smaller subset of articles (n = 274) of interest to this inquiry. Once we felt we had a strong comprehension of the breadth of the dataset, E.J.T. and F.M. then transitioned to the generation of initial codes from the data, where codes refer to the most basic segment, or element, of the raw data or information that can be assessed in a meaningful way regarding the phenomenon [49]. Next, we began identifying themes by sorting the specified codes (n = 8) and collating relevant data references (n = 616) [45]. For our analysis, we allowed coded data extracts to be assigned to as many different ‘themes’ as they fit into. We then refined and defined the themes by rereading all the collated extracts for each theme and considered whether they appeared to form a coherent pattern. We then evaluated the validity of each theme and whether it accurately reflected the meanings evident in the data set as a whole [45]. Finally, we chose to title the themes in vivo based on direct quotations from the media articles to reflect the original voices and situate the analysis in the reporting style rather than researcher-generated words and phrases [50] (Figure 1).

## 3. Results

Three main themes were identified within the articles that mentioned the 2021 Heat Dome and homelessness in Canada. The first theme highlights public warnings issued through the news media that directly addressed the heat vulnerability of people experiencing homelessness during the 2021 Heat Dome. The second theme captured content related to community support services that were activated to support this population, specifically during the heat event. The third theme then focused on the challenges and barriers faced by people experiencing homelessness during extreme heat that were communicated in the news media. The themes are descriptively presented below with quotations from the analysed news articles to illustrate the identified themes and analytical points. 

### 3.1. Theme I: “The Wave Could Create a Dangerous Situation for People Experiencing Homelessness”

Within the articles, there were various statements indicating that people experiencing homelessness are vulnerable to the heat, along with other population groups (e.g., elderly, children, chronically ill) (Box 1). Although less common, a few articles also mentioned vulnerability to compounding weather events or other environmental factors, such as ground-level ozone, which commonly co-occur with EHEs. For example, some articles referenced the Environment and Climate Change Canada heat alerts and air quality index risk levels, along with vulnerability warnings like “*Ground-level ozone is usually highest from midafternoon to early evening and can be particularly concerning for people with underlying health conditions and respiratory infections, such as COVID-19, as well as…people who do not have homes*”.

Box 1 **Heat-vulnerability statement examples**

“*Vulnerable people are more prone to heat-related death—especially the elderly, children, people living with chronic health conditions, individuals without housing, and low-income tenants who do not have adequate means to cool their homes during heat waves*”.

“*The people most susceptible to dying in extreme heat waves are people of colour, the elderly, people with underlying health conditions, mental illness or addiction, people who live alone, or in precarious housing and in units without adequate air-conditioning*”.

“*While everyone is at risk of heat-related illness, hot temperatures can be especially dangerous for the young, the elderly, those working or exercising in the heat, persons with chronic heart and lung conditions, persons with mental illness, people living alone and people experiencing homelessness*”.

“*If you’re in a very hot, small apartment or even worse, if you’re a homeless person, you’re much more at risk of suffering from illness during this extreme heat wave*”.

“*There is added concern for people who are living in substandard spaces and outdoors*”.

“*Try to mitigate the risk as much as possible, especially for people who are most vulnerable…those at higher risk include seniors, children, infants, people with chronic breathing or heart problems, people working or exercising outside and people without access to shelter*”.

“*It says exposure is particularly concerning for people with underlying health conditions and respiratory infections, such as COVID-19, as well as pregnant women, children, outdoor workers, older adults and unhoused people*”.

It was also common within the articles to articulate why people experiencing homelessness are vulnerable to the heat. For example, one article by a task force dedicated to ending homelessness in Burnaby, British Columbia, described that: “*The absolute homeless are vulnerable to severe health risks when hotter weather occurs for two or more days. Cities trap heat: on sunny days, pavement can be 27–50°—hotter than the air. Homeless people cope with unusual heat with minimal protection and have limited locations to access drinking water. Food spoils faster leading to higher risk of illness (i.e., botulism and salmonella). They face significant stigma, exacerbated by no or poor access to showers, laundry, or secure storage. They are often excluded from cooling off in air-conditioned malls and free public spaces. Many cope with pre-existing health conditions and all are at high risk of COVID-19*”. Many articles also spoke explicitly of this population’s lack of access to reprieve from the heat, especially at night, as temperatures did not subside during the 2021 Heat Dome. For example, one news article reported, “*During the week-long heat dome, temperatures did not cool off much at night, meaning there was little respite from the heat, especially for people living in sub-standard housing with no air conditioning*”.

A few articles also discussed the physiological strain placed on people experiencing homelessness in the heat, along with reports of heat-related illnesses and emergency medical service (EMS) deployments. For example, one article quoting an EMS services representative reported that “*[People] more directly affected by the heat that is repeatedly smashed same-day records this week have been those suffering heat exhaustion. Some of those patients are homeless individuals*”. Articles captured other heat-related conditions while interviewing community service providers, who reported: “*that people who are homeless or unsuitably housed were experiencing extreme sunburn and symptoms of heat stroke, something they fear will get worse as the summer continues*”. A few articles also referenced mortality outcomes for people experien–cing homelessness in the heat. For example, an interview between a reporter and Union Gospel Mission representative quoted that, “*Death is a possibility for those without shelter*”. Similarly, another article quoting a climate adaptation researcher stated that “*It’s not really the average person who’s likely to die from a heat wave event. It’s somebody who is living on the street, somebody who has pre-existing health conditions because they are not able to access the health care that they need*”.

Although most articles mentioned the health impacts as warnings, a few shared stories directly from people experiencing homelessness or insecure housing; for example, one article shared a series of excerpts from individuals living in an unairconditioned supportive housing unit near the Downtown East side of Vancouver, British Columbia, with quotes illustrating that “*it was so hot in the building that you could not even get your breath”.* Another occupant reported that the “*room[s] felt like a sauna, even at night. And when he went outside to scrounge for scrap metal, cans and bottles, he passed out multiple times from the heat*”.

### 3.2. Theme II: “Hot Weather Prompts Water Drive for Homeless”

The articles mentioned numerous community services being activated to support those experiencing homelessness. The most common initiative identified was the provision of water, with many articles helping to promote community agencies’ requests for bottled water donations. For example, one article reported: “*The wave could create a dangerous situation for [people] experiencing homelessness, worries that prompted the Mustard Seed to call for water bottle donations Wednesday after depleting their supply. It was an immediate, amazing, compassionate response*”. Many articles also reported on emergency services (e.g., peace officers, firefighters, paramedics) providing bottled water during the heat wave (e.g., “*Saskatoon police, fire departments handing out water amid heat wave*”). Likewise, social service groups reported similar initiatives. For example, “*This Salvation Army team is getting ready to head out again…We have our outreach team, who, on a daily basis, go out into the community and offer our unsheltered people water. Hydrate, hydrate, that is the message*”.

Groups went beyond bottled water in some areas to ensure broader water supplies were available. This included borrowing emergency water tanks (e.g., “*Alpha House also has teams handing out water and has borrowed an emergency water tank from the city to increase its supply of cold drinking water*”) and water wagons (e.g., “*In Calgary, water wagons were being deployed*”), as well as opening city buildings for water access (e.g., “*city centres were open to the public with water available*”). In addition, one article reported on a city installing additional water bottle filling stations, “*noting access to water is critical for those experiencing homelessness, who may spend lengthy periods outdoors in all types of weather*”.

Second to providing water, ensuring access to air-conditioned or cool spaces was the next most cited strategy in the media to support people experiencing homelessness during the 2021 Heat Dome. This included creating designated cooling centres and cooling stations. For example, “*Nanaimo has opened a cooling centre for people living on the street*” and “*Vernon organizations were quick to coordinate efforts to ensure everyone living rough or experiencing homelessness was able to access clean, cool water, showers and seek relief indoors at cooling stations*”. In addition, other agencies used the media to promote that their shelters had air conditioning. For example, “*At the Salvation Army’s downtown location, volunteers gave out free water and snacks to people. The shelter opened at all hours and has air conditioning available to patrons*”.

Other community groups were reported to have created additional shaded areas. For example, “*The Salvation Army in Chilliwack says it is providing more water resources at their shelter and have an area set up on their property for people who are homeless to access shade and water*”. Other groups established cooling tents (e.g., “*In Abbotsford, UGM [Union Gospel Mission] is working with other groups to set up two cooling tents for the homeless*”). Additionally, some agencies provided supplies for creating shade. For example, “*Be the Change YYC founder Chaz Smith said they will have teams downtown this week to distribute water and other necessary supplies like tarps, blankets and hats to create shade*”.

A few articles also mentioned ensuring that natural green spaces or parks were open for people experiencing homelessness to access shade. For example, “*The parks board planned to open the west side of the park by June 29…but decided to open it one day earlier than planned because of the heat wave*”. These articles commonly included statements reiterating the importance of the shade trees provide to people experiencing homelessness and how they “*help get people off the sweltering concrete*”. Other articles illustrated the effect of urban heat islands in areas without significant tree coverage, with one citing that “*On Saturday, it was 32 °C in the shade in the Downtown East side, a part of the city that gets hotter than other neighbourhoods because there are not as many trees to provide shade. It’s also the city’s poorest neighbourhood, where many residents are either homeless or living in poor housing*”. Another commonly mentioned strategy was establishing misting centres. Although all community members can access this strategy, it was often directly cited in media articles concerning homeless people. For example, one article stated, “*We have established two misting centres for homeless people as the mercury climbs. We put up the misting tents on days where there’s heat warnings like today…The canopy tent is equipped with hoses spraying a light mist into the air, and is stocked with a supply of drinking water*”. In addition to the core strategies of providing water and cool spaces, a few articles also mentioned support services providing people without housing with sun safety materials (i.e., sunscreen and hats), lighter summer clothing and cold consumables, such as freezies, Gatorades, and other cold foods.

Another strategy commonly cited in the media articles discussed outreach groups using different methods to provide people experiencing homelessness with heat-event relevant information, such as locations of cooling centres, where to find shade, which public facilities can be accessed during the day, where additional water resources can be found, and information on the dangers of heat. For example, one article cited, “*Outreach workers canvassed the town informing contacts that the cooling centre was available, and provided hats, sunscreen and water to those who visited*”. Another article reported that “*additional officers [had] been doing proactive patrols to reach vulnerable and unhoused community members to connect them with resources and provide information about three City of Burnaby cooling locations*”. In addition to the on-the-ground (or by-foot) strategies, other groups reported using print materials; for example, “*Staff at Our Place, which provides support for those who are homeless, have been putting up posters and warning those who are on the street of the dangers of heat exposure*”. This need to be ‘roving’ or moving for the population was frequently cited in the articles because, otherwise, the “*unhoused may not see messaging about how to find assistance*”.

Some communities also initiated ‘wellness checks,’ which included emergency services (e.g., police, firefighters) and other community outreach groups surveying the community on foot, bike or via patrol cars to conduct checks on the health and well-being of this population. For example, one article stated, “*Additional RCMP [Royal Canadian Mounted Police] members will be working in Burnaby this weekend making proactive patrols to check on vulnerable and unhoused people*”. Another cited that “*The city will also have outreach teams conduct wellness checks on people who live outdoors to provide them with water and recommend that they move to a shaded area if they stay outdoors*”. Although not discussed as much within the text, a few articles also mentioned homelessness outreach organizations monitoring and providing first aid when needed. 

Within the media articles, calls to the public to look out for the population living on the streets were also frequently identified. For example, one article stated, “*Edmontonians are being asked to keep an eye out for some of the city’s most vulnerable people who may be struggling as the province begins what is expected to be a historic heat wave…the heat can be dangerous for people who are homeless and he wants Edmontonians to keep watch for anyone who might be in trouble*”. In addition to broad public calls, other requests were published to private groups to help check on vulnerable groups (e.g., operators of single-room occupancy hotels).

Lastly, it was also identified in the media articles that some agencies also activated transportation services for people experiencing homelessness to help improve access to cooling centres or supply and service depots (provide basic services like food, drinking water, washrooms, sunscreen, clothing). For example, an agency called Pounds not only “*expand[ed] its hours during the heatwave to provide people with a safe place to avoid the heat…[but] also provided shuttle services and water delivery to and from known homeless camps in the community*”. Similarly, other agencies moved their services to high-density areas. For example, “*Normally, the Community Cares Kitchen…started providing those meals down at the riverbank too, along with cold water and other services…[as] many residents cannot make the trip to Access Place, even when temperatures are not climbing to near record levels*”.

### 3.3. Theme III: “Homeless People May Face Exclusion Due to Stigma from Cooling off in Air-Conditioned Spaces”

Additional challenges, barriers, and complexities experienced by people experiencing homelessness were often mentioned within the articles. Among the most common challenges reported was access to cooling centres outside of standard business hours. Although many groups established cooling areas (as discussed above), the limited hours of operation of some of these services posed a critical challenge. Many were reported to only be in operation Monday to Friday (i.e., not open weekends) and were open for limited hours. Further, many support services were unavailable on Canada Day (a statutory holiday), which fell within the week of the 2021 Heat Dome. Although some agencies could extend their hours during the heat wave and open cooling spaces over weekends, unhoused people had limited or no options for reprieve from the heat in some areas. 

Another noteworthy challenge faced by people experiencing homelessness in some areas during the 2021 Heat Dome was the removal of encampment communities (or ‘tent cities’), which provide shade to those without other options. For example, one article reported: “*Adding to the challenges are eviction notices that have gone up at two homeless encampments in the community…The city is warning people they have to leave the George Street camp Friday and the Patricia Boulevard camp on Monday, or face fines and arrest….About three dozen tents are in the encampment*”. Similarly, closures or the delayed opening of community parks posed challenges to the homeless during the 2021 Heat Dome, as illustrated by the executive director of the Overdose Prevention Society, who said, “*Keeping the park closed could have serious health consequences. Closing a park, having people lying around on the cement as an alternative could be lethal*”.

The COVID-19 pandemic was also ongoing at the time of the 2021 Heat Dome, which further exacerbated challenges for people experiencing homelessness. For example, one article quoted a community service provider saying, “*We’ve seen a lot of our clients with heat exhaustion, dealing with trying to stay hydrated, dealing with trying to find a cool place, which right now is really hard with COVID implications*”. The articles often attributed this to a lack of open cool spaces and less access to drinking water (e.g., “*People experiencing homelessness do not have access to fresh water like you and I do, and COVID has added an additional barrier to them even accessing public washrooms to find a tap*”). Further, COVID-19 public health measures, such as capacity limits, masking, physical distancing, and contact tracing (e.g., need for phone number or address), further complicated access to cooling facilities. For example, one article reported that the “*COVID-19 pandemic has added an extra obstacle because capacity restrictions in many buildings mean there are fewer indoor places to go to cool down*”. Similarly, COVID-19 protocols reduced access to other water features, such as fountains and spray parks. 

Another barrier identified was stigma towards those experiencing homelessness and its effect on accessing heat mitigation services. For example, one article cited that people experiencing homelessness “*may face exclusion due to stigma from cooling off in air-conditioned spaces such as malls and other publicly accessible facilities*”. Another article elaborated that despite drop-in cooling centres being offered at places like public libraries to alleviate the physical stress of constant heat, “*these environments can be uncomfortable and stigmatizing for people who are unhoused…I think the library does a good job of trying not to stigmatize, it’s just that walking into a place when you have not had a shower, and your option is to sit in a hard chair, and you’re tired but cannot fall asleep, and probably want to lay on the floor—it’s not ideal for friends without homes*”.

## 4. Discussion

Using a news media-based case study, we sought to identify potential actions or initiatives that may have supported people experiencing homelessness during the 2021 Heat Dome, as well as explore other implications of the heat event on this population. In doing so, we gained insight into individual actions and community-based strategies that were initiated to support people experiencing homelessness during this historic heat event. This included the broadcasting of warnings and statements for heat vulnerable groups and the activation of community support services (e.g., providing water, transportation). The articles also mentioned that the informal check-ins facilitated by enacting these services may also have assisted in protecting the members of this population from the heat. We also identified various challenges and barriers that people experiencing homelessness faced during the 2021 Heat Dome. The following subsections identify some of these gaps and translate these findings based on their implications for practice. 

Our findings demonstrated that throughout the region impacted by the 2021 Heat Dome, numerous community support services were activated to support people experiencing homelessness. Many of the most cited support services aligned with the literature on common behavioural adaptations to reduce heat stress among people experiencing homelessness, including spending time indoors in air-conditioned spaces, spending time outdoors in shady green spaces, wearing lighter clothing, and swimming [10,40,51,52]. Within our analysis, the most referenced support strategy was providing water, which included water bottles, borrowing emergency water tanks and water wagons, misting stations, opening city buildings for water access, and installing additional water bottle filling stations. This prioritization of water access aligns with the literature on general heat mitigation, and, more specifically, studies indicating that unhoused people have less access to safe drinking water [20,53,54]. There was also a focus in the media articles on supports that helped increase access to air-conditioned or cool spaces for homeless populations, which included creating designated cooling centres, cooling stations, cooling tents, or promoting shelters with air conditioning. Further, based on the reporting, there also appeared to be consideration granted for the location of some cooling centres in closer proximity to neighbourhoods with people experiencing homelessness. This, again, aligns with the literature, as it is well-established that unhoused people often shelter in accessible cooler spaces, including churches, shopping malls, public restrooms, and homeless shelters [11,18]. Although an array of services was captured in the media articles, they were dispersed across a large geographic area, with some jurisdictions only offering a smaller catalogue of options. To our knowledge, this study is the first academic publication to document the actions various communities have implemented to assist homeless populations during a major EHE in Canada. Further research could collate the array of potential planned and unplanned actions that other jurisdictions, both in Canada and beyond, have implemented to protect the health of homeless populations during heatwaves. This information could then help health officials and other service providers more strategically select and implement actions to protect homeless populations within their region. 

Through our analysis, we also found that many articles referenced community agencies creating additional shaded areas with tarps and tents, and providing supplies for shade building for people experiencing homelessness. Although providing tents and using material as shade is a strategy commonly used by unsheltered communities to reduce exposure from various elements, it may place individuals at further risk of adverse heat-related outcomes. A study by Karanja et al. [13] recently evaluated the efficacy of different tent cover (shading) materials and how they moderate the air temperature of tent users during extreme summer conditions. Their findings show that, in the daytime, the air temperature within a tent, whether covered or uncovered, is higher than the ambient air temperature; however, temperatures within a tent are lower at night [13]. Consequently, tents may not protect people experiencing homelessness from extreme summer air temperatures during the daytime and could exacerbate the risk of heat stress. Therefore, when community services are activating heat-mitigation initiatives during EHEs to support heat-vulnerable populations, such as those experiencing unsheltered homelessness, they could consider whether heat mitigation efforts further marginalize or exacerbate the vulnerability of this population [13]. Accordingly, service providers may consider assessing their current or proposed heat mitigation actions for unintended consequences. This would help inform the need for alternative strategies to protect those experiencing homelessness during EHEs. 

Content captured in the media also discussed locations for congregating/sheltering, as well as the removal of encampments during the 2021 Heat Dome. In some articles, content related to encampment removal included evidence of consideration for the extreme heat. For example, in Edmonton (Alberta), the normal encampment response protocol was paused during the extreme heat to focus on the immediate well-being of people in need. In contrast, in other locations, articles reported encampments being removed during the EHE but did not discuss the additional heat-related implications for the residents. As seen during other heat events globally, if evacuations or encampment removals are enforced, many unhoused people end up in hotter and less desirable locations, placing them at further risk [33,55]. Therefore, communities that do not currently address extreme heat within their encampment response protocols may consider how actions related to refuge destabilization during EHEs might put people at further risk of extreme heat and explore alternative solutions (e.g., shelters with air conditioning) [54]. 

Our findings also provided information related to access barriers for homeless populations that community service providers and public health authorities may consider when developing or revising their heat action plans in advance of future EHEs. For example, one of the commonly identified limitations was the restriction in hours for accessing cooling centres (e.g., weekdays only, standard business hours, no holiday hours). For example, one article referenced, “*some agencies [were] scrambling to open on Canada Day and over the weekend, fearing the heat could extend into next week. It’s just too hot to leave the community without support…This weekend is going to be extremely difficult because we’re also going into a holiday, so a lot of the organizations aren’t going to be open*”. The limited hours of the operation of cooling centres have previously been identified in other analyses and attributed primarily to staff and volunteer availability [18,54,56]. In acknowledging that a standard feature of EHEs is that nighttime minimum temperatures remain elevated [57], exploring the feasibility of extending the operational hours of cooling centres is warranted. Additionally, although less common, our analysis also highlighted that some areas launched additional transportation services for people experiencing homelessness to help improve access to cooling centres or supply depots. Other investigations have made similar recommendations to address the transportation-related challenges experienced by unhoused people [18,54,56], as the exertion required to transit to cool spaces can create unsafe conditions [58] and may cause financial burden. Therefore, future work could explore local barriers for people experiencing homelessness in using cooling centres to assist officials in better planning the location, hours, and accessibility of these services.

We found that outreach groups used different methods to provide people experiencing homelessness with heat-event relevant information, such as locations of cooling centres, where to find shade, which public facilities can be accessed during the day, where additional water resources can be found, and information on the dangers of heat. For example, some agencies physically moved their services to high-density areas, used on-the-ground (or by-foot) strategies (roving), or distributed print materials. However, as the literature highlights, communication with unhoused populations can be challenging, as they often do not receive municipal heat alerts [8,59]. Similarly, people experiencing homelessness may have inconsistent access to information (e.g., regarding cooling centres), as traditional word-of-mouth may facilitate misinformation [18,60]. Therefore, given there are various ways to provide heat-related information (e.g., mass media, posters, flyers, foot patrol) and it is unknown what the best mechanisms are for delivering messages to this population, future investigations could explore how to best communicate heat information with people experiencing homelessness. This could be pursued by directly contacting agencies that provided services during the 2021 Heat Dome, or other EHEs, to identify the communication strategies they employed to raise awareness of their heat-mitigation initiatives, as well as seek perspectives on the protective effect they may have had based on client feedback. Additionally, it is important to highlight that only a few of the online news articles analysed directly quoted people experiencing homelessness sharing their own experiences. Therefore, future studies may also consider exploring the actions that people experiencing homelessness self-initiate during extreme heat to ensure that future messaging and communication strategies are informed by lived experience [11]. 

We found that many of the vulnerability statements in the media articles included calls to the public from city and health officials to help look out for people experiencing homelessness, among other vulnerable groups. For example, one mayor was quoted saying “*I can’t emphasize enough the importance for us to look out for each other during this heat wave. We are especially concerned about our seniors and our citizens who are not able to escape the heat as they may not have air conditioning or suitable shelter*”. Acknowledging that traditional media may not always reach at-risk populations [8,59], and many unhoused individuals do not perceive themselves as vulnerable due to their past experiences navigating heat waves [18], community and peer support for health and wellness monitoring during EHEs is especially important. Further, a few articles also posted requests from city and public health officials to private groups to help check on vulnerable groups, such as landlords and operators of single-room occupancy hotels. Future studies could investigate if similar calls to action during EHEs, as well as the proposed actions themselves (e.g., wellness checks by landlords and other facility managers) are effective strategies for helping protect the heat-vulnerable population.

### Limitations and Future Studies

The limitations of our study indicate opportunities for future research. As this study is part of a larger investigation that reviewed and analysed online news media coverage on the 2021 Heat Dome, we only included articles from mass-media outlets, associations and agency press outlets specifically within Canada. Therefore, all international or social media-based sources were not explored. Thus, our findings reflect the interpretations of these sources as content generators, inclusive of their potential biases, and are limited to Canada’s news media landscape and may not reflect every North American region affected by the 2021 Heat Dome. Future investigations may consider exploring social media sources, as well as data which reflect the experiences in the areas of the United States similarly impacted by the 2021 Heat Dome. Further, it was not within the scope of this analysis to include content published by specific organizations, such as charitable organizations or religious groups which are active in supporting the community. Given the evidence from our media analysis that these groups were critical in supporting the community, future investigations may consider conducting a content analysis of their webpages and online resources related to EHE management and initiatives. 

## 5. Conclusions

Despite outdoor temperatures exceeding 40 °C in many areas during the 2021 Heat Dome and earning the title of the deadliest EHE recorded in Canada to date, people experiencing homelessness represented the smallest proportion of decedents. Indicative of underlying protection, we used online news media articles published in Canada to gain insight into the individual actions and community-based initiatives that may have supported people experiencing homelessness in surviving this historic heat event. We identified that the news media helped distribute public warnings that directly addressed the heat vulnerability of people experiencing homelessness during the 2021 Heat Dome. We also captured numerous community support services that were activated to support this population during the heat event. However, we also found that the news media highlighted various challenges and barriers faced by the people experiencing homelessness during extreme heat that were communicated in the media. With thousands of Canadians experiencing homelessness, and climate projections indicating an increasing trend of extreme heat intensity, magnitude, and duration, this could mean a higher predisposition for heat-related morbidity and mortality among the homeless [61,62]. Thus, the results of this study illustrate the need for public health interventions to consider critical factors of inclusion and drivers of differential health outcomes, which could help inform future research and improvements to support services for people experiencing homelessness.

## Data Availability

Data are available from the corresponding author (Glen P. Kenny, gkenny@uottawa.ca) upon reasonable request and signed access agreement.
